# Lessons learned from regional training of paediatric nephrology fellows in Africa

**DOI:** 10.1007/s00467-023-06022-9

**Published:** 2023-06-06

**Authors:** Mignon I. McCulloch, Andrew C. Argent, Brenda Morrow, Peter Nourse, Ashton Coetzee, Christel Du Buisson, Deveshni Reddy, Jonathan Buckley, Paul J. Sinclair, Priya Gajjar, Lucie Semanska, Allison Eddy, John Feehally, Francisco Cano, Bradley A. Warady

**Affiliations:** 1 Department of Paediatric Nephrology, Red Cross Children’s Hospital, University of Cape Town, Rondebosch, Cape Town, South Africa; 2 Department of Paediatrics and Child Health, University of Cape Town, Cape Town, South Africa; 3 Department of Paediatric Nephrology, Tygerberg Hospital Children’s Hospital, University of Stellenbosch, Stellenbosch, South Africa; 4 International Pediatric Nephrology Association (IPNA) Office Administration, IPNA Programs Coordinator, Kansa City, MO USA; 5 Professor Emeritus (Pediatrics), The University of British Columbia|Musqueam Traditional Territory, Investigator, BC Children’s Hospital Research Institute, Scientific & Research Staff, BC Children’s Hospital, Vancouver, Canada; 6 Emeritus Consultant Nephrologist, University Hospitals of Leicester, Honorary Visiting Fellow, Leicester, UK; 7 Pediatric Nephrologist, Luis Calvo Mackenna Children’s Hospital, University of Chile, Santiago, Chile; 8 Division of Nephrology, Childrens Mercy, Kansas City, MO USA

**Keywords:** Kidney disease, Pediatric nephrology fellows, Training, Hands-on, Acute kidney injury, Dialysis

## Abstract

**Background:**

Access to care for children with kidney disease is limited in less well-resourced regions of the world and paediatric nephrology (PN) workforce development with good practical skills is critical.

**Methods:**

Retrospective review of a PN training program and trainee feedback from 1999 to 2021, based at Red Cross War Memorial Children’s Hospital (RCWMCH), University of Cape Town.

**Results:**

A regionally appropriate 1–2-year training program enrolled 38 fellows with an initial 100% return rate to their country of origin. Program funding included fellowships from the International Pediatric Nephrology Association (IPNA), International Society of Nephrology (ISN), International Society of Peritoneal Dialysis (ISPD), and the African Paediatric Fellowship Program (APFP). Fellows were trained on both in- and out-patient management of infants and children with kidney disorders. “Hands-on skills” training included examination, diagnosis and management skills, practical insertion of peritoneal dialysis catheters for management of acute kidney injury and kidney biopsies. Of 16 trainees who completed > 1 year of training, 14 (88%) successfully completed subspecialty exams and 9 (56%) completed a master’s degree with a research component. PN fellows reported that their training was appropriate and enabled them to make a difference in their respective communities.

**Conclusions:**

This training program has successfully equipped African physicians with the requisite knowledge and skills to provide PN services in resource-constrained areas for children with kidney disease. The provision of funding from multiple organizations committed to paediatric kidney disease has contributed to the success of the program, along with the fellows’ commitment to build PN healthcare capacity in Africa.

**Graphical abstract:**

**Figure:**
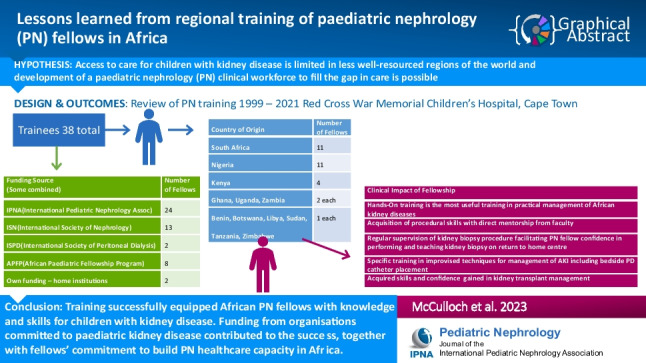
A higher resolution version of the Graphical abstract is available as Supplementary information

**Supplementary Information:**

The online version contains supplementary material available at 10.1007/s00467-023-06022-9.

## Introduction

Access to care for adult patients with kidney disease is a challenge in less well-resourced communities [1] around the world and this situation is particularly bad for African children and adolescents with kidney disease, for whom even basic management resources for treatment of acute kidney injury (AKI) is often not available [2]. Regions in Sub-Saharan Africa, with an estimated population of 430 million children in 2015, face the same increases in demand for health services to address the “triple burden of disease” (communicable and non-communicable diseases and injury) along with extremely challenging social determinants of health (e.g., undernutrition; lack of access to education; problems related to globalization) [3].

A well-trained healthcare workforce is critical to optimize healthcare in less well-resourced countries and to achieve the sustainable development goals. In this context, training a paediatric specific nephrology workforce is critical to meet the increasing worldwide burden of kidney disease [4].

The availability of nephrologists who are able to provide care for individuals with kidney disorders is much higher in high income countries (HIC) compared to low income countries (LIC) [4]. Riaz et al. [5] reported a global nephrologist density of 10.0 per million population (pmp) and a nephrology trainee density of 1.4 pmp; however, the distribution of these human resources is highly variable, with LIC reporting nephrologist and nephrology trainee densities of 0.2 pmp and 0.1p mp compared to HIC densities of 23.2 pmp and 3.8 pmp, respectively. African and South Asian regions do not have a workforce that is sufficient to meet current clinical needs or one that is sustainable with the potential for population growth. A recent publication [6] has predicted an insufficient number of adult nephrologists globally by 2030, unless there is an increase in funded training posts and posts for qualified nephrologists in the public sector. The situation in paediatric nephrology is even more concerning [7]. The shortages in the nephrology workforce can be attributed to many factors, including limited physician training capacity [8] and migration of skilled workers across and between regions [9].

Various organizations have attempted to address some aspects of this problem by training nephrologists for adult practice. For instance, as part of its building capacity and outreach initiative, the International Society of Nephrology (ISN) has a fellowship program in which trainees travel to an advanced nephrology center to obtain skills and training and then return to their home country to practice [10–12]. In addition to enhanced patient care, positive impacts of the fellowships have included continuing medical education (CME), sister kidney centers (nephrology units in emerging economies teaming up with a supporting center), and clinical research educational ambassadors (receiving expert guidance and teaching) [10].

Physicians from low- and middle-income countries (LMIC) who undergo paediatric and adult nephrology training in high resource areas such as North America and Europe, however, often face many challenges upon returning to their countries of origin. These can include an inability to apply what they learned or experienced as a result of differences in disease patterns, poor availability of equipment, as well as a lack of support from management, administration, and even colleagues [10, 13, 14]. For example, there may be a lack of paediatric nephrology (PN) training in the management of endemic conditions such as AKI due to gastroenteritis and sepsis, and clinical skills are usually taught using highly specialized equipment and consumables that are not available in many African centers. In addition, due to regulations surrounding registration for clinical practice, many fellows training outside of Africa, cannot participate fully in “hands-on training” including performing kidney biopsies and bedside insertion of acute peritoneal dialysis (PD) catheters and hemodialysis (HD) lines. Following training, many frustrated doctors may, in turn, not return to their countries of origin, further compromising the nephrology workforce.

Currently, South African regulations allow limited registration for fellowship trainees, which enables “hands-on” clinical practice under supervision in training institutions such as the Red Cross War Memorial Children’s Hospital (RCWMCH), situated in Cape Town. This center has developed a number of inexpensive and readily available improvisational interventions and devices that can be taught to African trainees to apply within their home settings [15, 16]. These include the use of multipurpose catheters, central lines, and chest drains, or nasogastric tubes for peritoneal dialysis (PD) access, in addition to locally made PD fluid [17, 18]. In the early 2000’s, the International Pediatric Nephrology Association (IPNA) approved Red Cross War Memorial Children’s Hospital (RCWMCH), University of Cape Town (UCT), as one of the first training centers for paediatric nephrology (PN) in a less well-resourced country and committed to funding a number of individual 6 to 24-month nephrology training fellowships. The ISN and the International Society for Peritoneal Dialysis (ISPD) have subsequently also assisted in providing pediatric nephrology fellowship funding, despite being predominantly adult programs. Very little has been published about paediatric nephrology fellowship training as a strategy to expand the nephrology workforce in LMIC. This paper aims to describe a paediatric nephrology training program within an African setting. Most of the fellows described in this paper originate from countries where AKI is common due to acute diarrhoeal diseases and infections including malaria. Chronic kidney disease (CKD) is common in their countries as a result of undiagnosed congenital kidney pathology due to a lack of antenatal screening and the poor availability of genetic services. In addition, chronic dialysis and transplantation has only been an option for children in a few countries in Africa. Finally, with the exception of South Africa, most of the other Sub-Saharan African countries require self-funding by families for the care of patients with paediatric kidney diseases, but prior to our training program, almost no pediatric nephrology services were available in Sub-Saharan Africa.

## Materials and methods

This paper presents a descriptive audit of the development, curriculum, funding support, outputs, and experience of a paediatric nephrology (PN) fellowship training program between 1999 and 2021.

### Study site

The training program was initiated and largely conducted at the Red Cross War Memorial Children’s Hospital (RCWMCH), Cape Town, South Africa. RCWMCH is a dedicated tertiary level, 300-bed paediatric hospital with a 12-bed paediatric nephrology and transplant unit providing care for 400 in-patients per year and 2000 outpatients. The hands-on training includes instruction on kidney biopsies, bedside insertion of acute PD catheters and all forms of acute and chronic kidney replacement therapy (KRT) for patients ranging from infants to adolescents in the nephrology unit, as well as in the 30-bed paediatric intensive care unit and neonatal high care. Senior nephrology staff consisted of 2–3 consultants over the study period, who provided all training. Whereas most of the training occurred at RCWMCH, training also occurred at the PN Unit at Tygerberg Hospital/University of Stellenbosch and in the adult tertiary center, Groote Schuur Hospital (GSH).

RCWMCH and GSH started an adolescent nephrology service in 2002, which was a relatively new concept and is still the only one of its kind in South Africa. Our fellows were also able to spend some time in this clinic learning how to manage adolescents with kidney problems and how to transition what they learned upon their return to their home institutions. We would hope that this would inspire paediatric and adult nephrologists to work more closely together (as occurs in ISN) and to set up similar programs with comparable content across Africa (context of training in paediatric nephrology training Supplemental Appendix 1). In addition, 5 adult nephrology trainees from the GSH Adult Nephrology Department spent 1 month in the pediatric training program at RCWMCH following the completion of their adult nephrology sub-speciality examinations. This was intended to provide them with skills to manage children with kidney diseases in addition to their adult patients upon return to their home institutions in regions where paediatric nephrology care was not available.

### Participants

All individuals who participated in PN training were included in this review. For acceptance into the training program, fellows had to meet well-defined criteria including having completed training in general pediatrics; no prior formal training in pediatric nephrology; and approval of the application by the IPNA fellowship committee.

### Data collection and analysis

Data obtained include details of all individuals who participated in PN training at the study site, including gender, country and institution of origin, period of training, and whether any PN tertiary qualifications were achieved. All African PN fellows from outside South Africa also completed a survey on completion of their training (Supplement Appendix 2).

The themes addressed included pre- and post-training work opportunities, and areas of training that were deemed particularly useful, including specific skills and exposure to multidisciplinary teams. Experiences with hands-on training opportunities were also explored.

### Funding

Funding for the training program originated from three main sources: the IPNA and the ISN, both of which provided 1–2 years of funding per trainee, and the ISPD which provided funding for shorter durations of training (3 months).

Locally, the African Fellowship Program/Children’s Hospital Trust (APFP/CHT) provided substantial administrative and moral support for all fellowship programs, as well as additional funding in cases where there was a shortfall.

### Ethics

This study complied with the ethical guidelines and principles of the Helsinki Declaration of 2008, South African Guidelines for Good Clinical Practice, and the MRC Ethical Guidelines for Research and was approved by the Human Research Ethics Committee of the University of Cape Town (Ethics: 646/2015). Fellows completing the questionnaire consented to the publication of the information provided.

## Results

### Training content

All fellows received comprehensive training in theoretical and clinical/practical aspects of paediatric nephrology, complemented by hands-on clinical training with prioritization of the management of AKI. A formal syllabus and weekly timetable were developed to address the educational content of the fellowship experience (Supplement Appendix 3). Fellows could attain paediatric nephrology levels 1 and 2 training competence based upon the training content (Supplement Appendix 4).

Ultimately, there is potential for the development of a common paediatric kidney training syllabus across the African continent.

### Study population and demographics

A total of 38 paediatric nephrology fellows were trained at RCWMCH from 1999 to 2021. Eleven (28.9%; 5 male) fellows were from South Africa (SA), with all but one funded by local provincial funding. Eight of the SA fellows were from the Western Cape province and three were from other SA provinces. All completed 2 years of training and achieved their PN sub-specialty accreditation. All but three SA fellows returned to their original centers, two of whom are working in private practice due to a shortage of public hospital posts, but remain as honorary lecturers for UCT/RCWMCH. Twenty-seven (71.1%; 14 male) fellows were from twelve anglophone African countries outside SA. The country of origin of the fellows is listed in Table 1.

**Table 1:** Fellows country of origin

Country of origin	Number of fellows
South Africa	11
Nigeria	11
Kenya	4
Ghana	2
Uganda	2
Zambia	2
Benin	1
Botswana	1
Libya	1
Sudan	1
Tanzania	1
Zimbabwe	1

The program has trained an average of 2 PN fellows per year overall, with the number increasing to 3 in the last 5 years. A total of 28 fellows from Africa have been trained (Supplement Appendix 5). The sources of funding for training are described in Table 2. The PN fellows from outside SA (African fellows) have had varied sources of funding, with IPNA and ISN supplying the majority of funds.

**Table 2:** Funding sources for training

Funding source	Number of fellows*
IPNA (International Pediatric Nephrology Association)	24
ISN (International Society of Nephrology)	13
ISPD (International Society of Peritoneal Dialysis)	2
APFP (African Paediatric Fellowship Program)	8
Own funding	2 (Komfo Anokye Hospital, Ghana; Beit Fellowship Zambia)

^*^Some fellows were dual-funded

### Impact of COVID

During the worldwide COVID-19 pandemic, the PN fellows continued their training at the RCWMCH uninterrupted. They were educated about the risks of COVID, provided with the required personal protective equipment (PPE) and afforded the same medical facilities as the local doctors, including access to COVID-19 testing and vaccines. The presence of COVID-19 did, however, preclude travel to their countries of origin for prolonged periods of time and compromise any short rotations to the adult hospitals, potentially impacting on the clinical experience gained in adult nephrology.

### Duration of training

The duration of fellowship training for the African PN fellows varied, largely dependent on available funding. The training period was divided into two consecutive periods for analytical purposes: 2003–2012 (period 1) and 2013–2021 (period 2). Twelve African PN fellows were trained in the first period and 16 in the second period. During the first period, African PN fellows trained for a shorter duration with a mean of 11.9 (median 12) months; while training during the second period was longer with a mean and median training time of 18.4 and 23 months, respectively. The duration of training for the African Pediatric Nephrology fellows is documented in Table 3**.**

**Table 3:** Duration of PN training for African PN fellows

Length of time (months)	Number of fellows	
3	1	12 months or less (*n* = 12)
6	5	
12	6	
15	3	15–24 months (*n* = 16)
18	5	
24	8	

### Qualifications achieved

Qualifications obtained by PN fellows during training included a post-graduate diploma in PN (University of Cape Town 1-year program) or a sub-specialty certificate in pediatric nephrology (College of Paediatrics of South Africa – USA board certification equivalent), which required at least 18–24 months of supervised training at RCWMCH to be eligible to sit for the written, oral, and OSCE exam. Many of the African PN fellows now require a recognized qualification to return to their home institution for positions of leadership either at their universities or their hospitals. Of those fellows who completed ≤ 12 months training, only 3/12 (25%) completed a diploma in PN, whereas 14/16 (88%) fellows training for > 15 months graduated with a sub-specialty certificate in PN.

African PN fellows training at RCWMCH for more than 12 months can register with the University of Cape Town for a Master’s in Philosophy degree, which uses the College of Paediatric Certificate as the clinical examination and requires an additional research component.

Nine (56%) African PN fellows, all of whom trained for > 18 months, achieved this MPhil degree, re-affirming that 18 months was the minimum time required for optimal clinical and research training.

### Research

Research is encouraged as part of the training, but has not always been possible for short fellowship training periods, as acquisition of hands-on clinical skills takes precedence.

Fellows who received more than 1 year of funded training were able to engage in research including topics such as patient audits of posterior urethral valves, pelvi-ureteric junction obstruction, crescentic nephritis, vitamin D status in CKD, drop out from chronic peritoneal dialysis, aminophylline affect on urine output, tuberculosis in paediatric kidney transplants, review of urodynamic studies, acute post-streptococcal glomerulonephritis, and severity of deranged electrolytes and kidney function.

### Social factors

A majority of the fellows (*n* = 25; 92.6%) were unable to see their families for the full duration of training. Only three of the fellows were able to bring their families to Cape Town with them. This is evidence of the commitment and dedication the fellows had with respect to learning and the provision of service to children with paediatric nephrology disorders in their home countries.

### Fellowship follow-up

All 27 (100%) of the African PN fellows initially returned to their countries of origin for at least 2 years, supporting the concept of training “in Africa for Africans.” Subsequently, two PN fellows emigrated—one went to the Middle East in view of an unstable political situation in their home country, and the other moved to the UK as their partner was transferred there. Thus, 94.7% of trained African fellows remain in their home countries.

### Post-training survey results

A voluntary survey was sent to all PN fellows coming from outside of South Africa following the completion of their training with a 100% (28/28) return rate (Supplement Appendix 2). Work positions changed in all cases to a more senior position once fellowship training was completed with a number becoming heads of departments in their hospitals (*n* = 4), lecturers in their universities (*n* = 18), and vice dean (*n* = 1).

### Time dedicated to paediatric nephrology

Upon completion of fellowship training, the percentage of work effort dedicated to PN varied, with > 60% spending > 50% of their time in clinical PN (Table 4) More detailed information regarding daily workload on return to home institutions including clinical, teaching, and administrative commitments is available in Supplement Appendix 6.

**Table 4:** Time spent in clinical paediatric nephrology

Number of fellows (%)	Percentage of total work time
4 (16%)	75–100%
12 (48%)	50–75%
4 (16%)	25–50%
3 (12%)	10–25%
2 (8%)	< 10%

### Work facility

The majority of PN fellows worked in state or university hospitals following the completion of their training (Supplement Appendix 7)**.**

### Institutional support

Upon completion of their PN fellowships and return to their countries of origin, 12 (50% fellows who completed this part of the questionnaire) received excellent institutional support, 10 (42%) received some support, and 2 (8%) received no support. Paediatric kidney facilities available on return from training was variable (Table 5).

**Table 5:** Paediatric facilities available on return

	Frequently available	Occasionally available	Rarely available
Equipment	Adult PD and HD catheters	Paediatric HD lines	Paediatric PD, HD, and consumables
Machines	Ultrasound machines Adult dialysis machines	Paediatric dialysis machines—HD	Paediatric cycling PD machines
Radiology	By day	At night or on weekends	Nuclear medicine and Urodynamics
Histology	By day Light microscopy	At night or on weekends	Electron microscopy/immunohistopathology
Surgeons and urologists	Mainly adult trained (65%)	Paediatric surgeons (30%)	Paediatric urologists

### AKI management

**Fig. 1:**
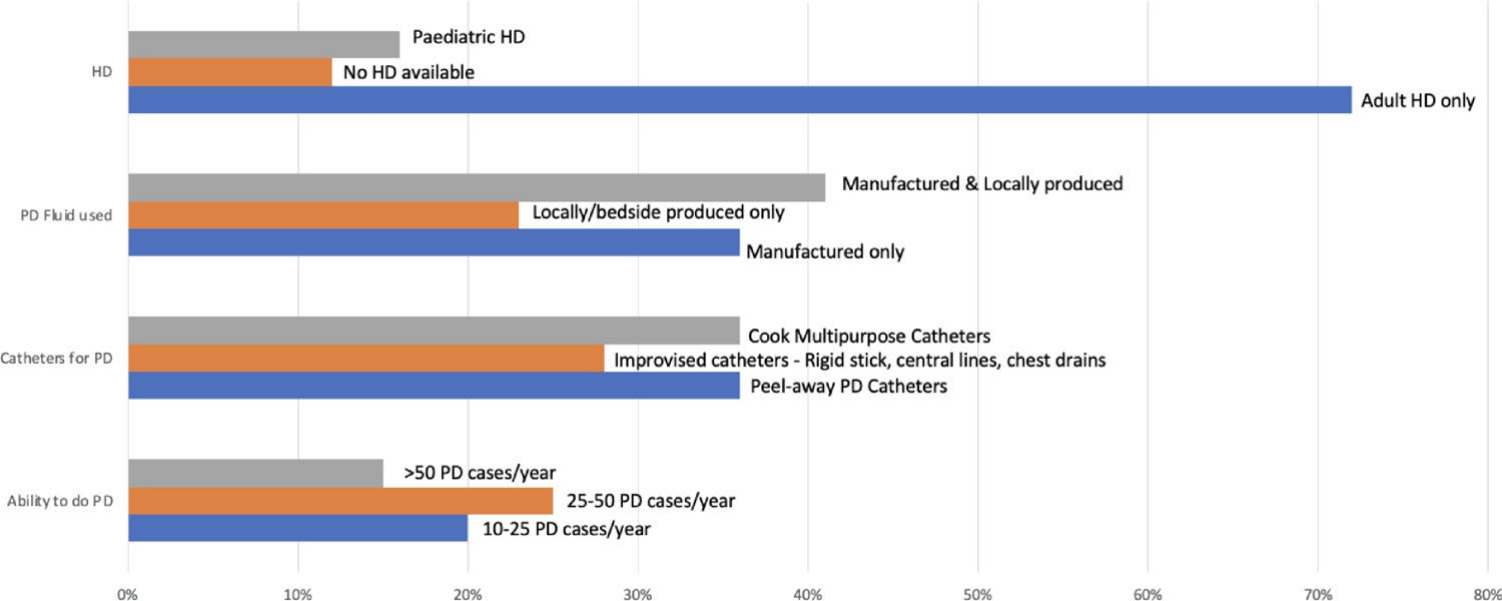
AKI management and resources in home institutions. The dialysis modalities used, the supplies available and the patient volumes were variable. HD, hemodialysis; PD, peritoneal dialysis

PD remains a challenge in view of lack of dialysis solutions and standard PD catheters, resulting in the need to train fellows to have improvisation skills with homemade fluids and makeshift catheters (Fig. 1).

### Community health care

There was unanimous agreement that health care in the local community had been positively affected by PN training and the return of the trained fellow to their home institution. Qualitative comments pertaining to the impact of PN training were collected (Table 6).

**Table 6:** Impact of PN training on community health care

*Majority of patients are poor and can’t afford investigations, lack of equipment*
*Accumulation large numbers of CKD patients with no dialysis or transplantation access*
*Set up lots of firsts/lots of acute PD, transplant, biopsies and fellowship training*
*Improved care for renal problems in children*
*My training has had a positive impact in my community, revised pediatric nephrology curriculum for medical school and post graduates; also our protocols, regularly talks on Ped Neph topics resulting in many difficult patients referred and phone consultation for difficult remote cases…also trained a team (doctor* + *nurse) from DRC on hands-on PD; who are doing very well*
*Yes. My unit has trained general medical officers in performing emergency PD for AKI. Regular consult from district*
*The training we pass on to our undergraduate and also during CME was significantly improved early diagnosis and referral of patient that require specialized care. Besides, understanding of preventable renal condition has significantly improved among doctors*
*Largely. One of two paediatric nephrologists in a city of about 20 million; median age is about 18 years*
*Improved identification of CKD, early intervention for acute kidney injury and better control for nephrotic children*
*Established enuresis clinic which serves a large population, contributed to establishing a charity for children with renal disease and contributed to HD unit and plasmapheresis*
*More nephrological problems are definitely being identified with better structure to patient management protocols and training doctors and nurses from different parts of the country in clinical skills*
*Yes—now have knowledge and capacity to do PD*
*Recognition of AKI with improved mortality and introduction of dialysis at the children’s hospital and also improving picu capacity. Improved protocols of nephrotic syndrome* *Established first paediatric nephro-urology collaboration in country to diagnose and integrate care. Established only paediatric chronic pd program in the country and train*
*Very much so, there has been an improvement in the care of children with kidney disorders in Tanzania, Telenephrology services for other centers which are far from my center. Yes, management of children with kidney diseases have changed with my training, due to better and more organized care, e.g., paediatric NS not responding to steroid, now biopsied and receive immunosuppressants (CNI/MMF)*
*Identifying nephrological problems, YES. New approaches to management of renal issues, YES, training ongoing, long distance phone consults also happen. Clinical training, people skills, administrative and management issues have and continue to change*
*More healthcare professional have been educated on management of paediatric renal conditions and this has translated into better clinical outcomes*
*More nephrological problems identified—more recently lupus nephritis. Paediatric KRT for AKI done- though sometimes takes a lot of effort in terms of adaptations (a bit more difficult to teach others), as procedure done manually. Despite challenges we have seen some remarkable recoveries from AKI. Awareness and detection of pediatric AKI is high. Number of cases that are diagnostic challenges are also becoming less*
*We are picking more CAKUT, especially PUVs* *Some doctors are able to insert acute PD*
*Yes there has been a change as people are becoming more aware of Paediatric renal conditions and we are getting calls from across the country regarding management of various renal conditions and whether or not to refer to the unit*

### Teaching

All of the African PN fellows were required to provide teaching for both undergraduate and post-graduate students upon returning to their institutions. When asked if PN training prepared them for teaching, 12 (46%) felt they were well prepared to teach, 3 (12%) felt they were prepared to some extent, 2 (7%) felt they were not prepared, and 9 (35%) did not respond to this question. In general, fellows who stayed for a longer duration of training felt more confident with teaching.

### Benefits of the training program

Overall, hands-on training was found to be very valuable and was deemed to be the most useful part of the training program on completion (Table 7).

**Table 7:** Feedback from fellows on completion of training program

Hands-on training
Most useful part of the training given opportunities to gain appropriate skills
Allowing for practical management of common kidney diseases in Africa
Doing on-calls with mentor support
Procedures
Under close supervision giving confidence in kidney biopsies, placing bedside PD catheters, and setting up PD and forming the basis of what fellows do and giving an ability to train others
Doing procedures meant that you cannot forget what you saw and exercised (practiced) as to do a procedure oneself is more effective than just watching it being done
Where one makes mistakes, the person is immediately corrected by the trainer, and gets the opportunity to do it again, thus acquiring the skill. If it were only an observership, the trainee will not benefit from the knowledge inherent in practical errors
Acquiring Skills
Acquiring skills while, e.g., renal biopsy being careful watched which allowed for correction of technique as well as a safe space to practice while feeling assured that there was someone who “had your back”
The training was also felt to be appropriate for the setting that the fellows were returning to in Africa and teaching adapted or improvised techniques of managing AKI
Specific training
Spending time in the HD unit provided knowledge and confidence to establish HD for children
Confidence was also gained in making decisions in managing transplant patients
Visiting other units while training
Our combined sister platform Paediatric Nephrology Unit at Tygerberg Hospital/University of Stellenbosch (TBH/US) for varying periods of time as well as telephonic on-call support
Short periods (1 month) were also spent at our adult unit Groote Schuur Hospital (GSH), but this was curtailed during the Covid pandemic

### Modifications to the training program

The majority (16/28) of the fellows who were able to spend more than 1 year in training managed to obtain enough experience in conducting kidney biopsies, acute PD, HD (lines and technique), KRT, and research time. Although the program was deemed to be comprehensive, the fellows reported that they required a longer duration of training to conduct research or learn teaching skills. Overall, a program lasting 18–24 months was seen as the ideal time period to learn all that was required. Additional recommendations for improved training are provided in Supplement Appendix 8.

### Assessment of the training experience

All (100%) of the fellows felt that they would strongly recommend the program in terms of its hands-on approach and acquisition of skills. Subjective feedback from fellows on their training experience with subsequent recommendations has been collected (Supplement Appendix 9).

## Discussion

The attractiveness of nephrology as a specialty has diminished over the past few decades leading to global concerns regarding the future of the specialty’s workforce, even more so in LMIC [11]. There has been a call to boost recruitment of both adult and paediatric nephrologists by increasing exposure of medical students to nephrology, providing mentoring, improving the clinical experience, incorporating procedural skills, facilitating exchanges between trainees and senior nephrologists, adapting active approaches to identify dissatisfaction and burnout, increasing renumeration, and incentivizing advances in the field of nephrology [12].

Some high-income countries rely on foreign-trained doctors to cover shortages in nephrology staffing. For example, a study from Oman showed that the majority of practicing nephrologists were expatriate physicians, with local doctors representing only 14% of the workforce [13]. In the USA, a recent report showed that international medical graduates represented 47% of active nephrologists and 65% of nephrology trainees [14]. By comparison, our fellows had a 100% return rate to their home countries reversing the “brain drain” and providing PN knowledge to these countries.

The Paediatric Nephrology Department at RCWMCH/UCT and the APFP/CHT have, with funding from international nephrology organizations, been able to assist in regional training of “African paediatric nephrology fellows in Africa for Africa” in 1 center. Invaluable experience was gained with “hands-on” patient examinations, diagnosis, and management, as well as procedures. Peritoneal dialysis for management of pediatric AKI using adapted techniques in the absence of the availability of a paediatric surgeon to place a Tenckhoff in a theater facility, has now become accepted as a safe and effective alternative, as recently published in the updated ISPD guidelines for management of children with AKI [19].

In the first decade of training, most of the fellows stayed for 1 year or less; however, the training duration of the fellows in the second decade increased to 2 years as home institutions requested that their fellows complete training with formal qualifications. As trainers, we also strongly supported this philosophy in keeping with international norms of 2–4 years of nephrology training [20] which often includes some clinical research training, the next logical step for our fellows following clinical training. In some cases, PN fellows have returned to their home institutions without completing their research training and then found it very difficult for them to complete it, as they have lacked clinical support to assist them in meeting the large workload of patients.

The measure of success of this program, in addition to the fellows acquiring sub-specialty exam/post-graduate qualifications or master’s degrees, is the fact that there has been an initial “100% return rate” at 1 year to home institutions in Africa. The training program tried to ensure that the referring institution was prepared to support trained fellows and offer them a position upon their return home. This is an area where human healthcare resource planning can become more involved in local countries.

More than two-thirds of the fellows returned to government/university positions, on occasion being part-time for those employed in private hospitals. As medical directors of kidney/dialysis units, communication skills, staff empowerment, allocation of resources, mentoring, team building, and strategic planning are all important skills [21, 22] and they learned these skills while training at RCWMCH. On return to their home centers, many of the fellows had their skills recognized which enabled them to take up more influential positions as heads of departments within their hospitals and universities.

In terms of daily workload, the majority of the fellows spent their time post-training in clinical work as opposed to teaching, with very little time dedicated to administration. Unfortunately, the survey did not specifically ask about dedicated research time or whether this was included in clinical or administration time.

Despite the recent publication of ISPD guidelines pertaining to the use of PD for management of AKI, overall there has been a decline in the use of PD in high-resource countries and this has resulted in the loss or absence of knowledge on PD leading to an unwarranted pessimistic view of this form of KRT [23, 24]. On review of the survey data pertaining to dialysis training for AKI and especially PD training with hands-on PD insertion techniques, the fellows generally felt it to be “extensively covered” and resulted in fellows being able to perform PD for the management of AKI in their own centers and characterizing it as a center strength. The benefits of PD in this setting have also been experienced by the “Saving Young Lives” initiative. Challenges pertaining to the provision of PD in LMICs include lack of PD catheters, consumables, and PD solutions [25]. PN working in these regions have highlighted their needs to focus on pragmatic pathways to provide kidney support therapy. This program has provided that particular focus and it has been recognized as a strength by the fellows.

In general, “hands-on training” was found to be the most useful part of the training, providing opportunities for fellows to acquire the skills necessary for practical management of common kidney diseases in Africa, as well as meeting on-call demands with mentor support. Supervised training for procedures such as kidney biopsies, placing bedside PD catheters, and setting up a PD system form the basis of what nephrologists do and promotes the training of others who often practice without the backup and support of interventional radiologists and surgeons specifically trained in pediatrics.

On return to their home institutions, 50% of fellows felt that they did receive some support from their institutions, but the remainder felt that they needed significantly more. The most useful equipment included dialysis (PD and HD) and biopsy consumables and ultrasound machines. Other departments thought to be essential, but with limited time availability and resources included radiology (only office hour availability and absence of nuclear medicine and urodynamics), histology (office hours only and lack of EM and immunohistochemistry) and surgeons (shortage of paediatric trained surgeons and urologists, in particular). The role for training opportunities for allied and nursing healthcare workers (e.g., dialysis nurses, dialysis technicians, dietitians, and others) in their native languages also needs to be addressed because of their essential role in pediatric kidney care.

Despite the challenges in their institutions, the fellows felt unanimously that their training had enabled them to positively affect health care in their community with a summary statement illustrating this: *Yes, there has been a change as people are becoming more aware of paediatric kidney conditions and we are getting calls from across the country regarding management of various kidney conditions and whether or not to refer to the unit.*

An important part of their training was in the field of advocacy for children and adolescents with kidney disease.

Whereas data collection with identification of patient outcomes and a review of the impact of training and resources on outcomes in LMIC is difficult, registries of kidney disease in children need to be established in Africa, as occurs with other adult and pediatric kidney patients to determine the true extent of children’s kidney disease and to facilitate the generation of successful diagnostic and treatment strategies [26–28].

Teaching of both under- and post-graduate students was an essential part of the fellows’ responsibilities and in general, fellows who stayed for longer training, overall felt more confident with teaching. Whereas CME was also an essential part of training with many feeling it was adequate, suggestions have been made specifically to develop patient treatment pathways relevant to local conditions. In addition, the role of virtual webinars as a widely available avenue for CME education throughout LMICs requires close attention and evaluation.

Recommended modifications to the program include recommendations that the program duration be a minimum of 18–24 months to allow for sub-specialty exams in PN and to permit acquisition of skills in HD (iline access and chronic HD), KRT in PICU, teaching, and research methodologies, with time to complete any research projects once they have started.

Additional recommendations included development of different levels of training to allow for some local training followed by concentrated training in advanced nephrology once enough PN units have been established in the region, as well as post-graduate networking among fellows to promote the development of sister center programs. Many fellows are already on a WhatsApp group as a form of promoting a “PN Fellowship Network” in Africa and this support should be extended further (Table 8).

**Table 8:** Recommendations arising from this paper for a pediatric nephrology training program

RCWMCH as a training center has been acknowledged as appropriate and excellent in terms of “hands-on training.” However, 6–12 months is too short and ideally need 18–24 months for “hands-on training,” teaching and research Training to include case presentations on ward rounds as well as on-calls with senior backup Also, Cape Town is expensive in terms of accommodation and travel and thus adequate funding is required to manage financially in this center and allow visits home
Extra time spent at this training center in addition to AKI and CKD management and dialysis as well as transplantation, would benefit in learning advanced techniques of HD including line insertion and managing HD/CKRT in PICU settings. This is accommodated in a longer training program more than 12 months
Training in urodynamic studies and assessments
Training in developing registries—in less well-resourced countries**,** few adult units have registries but it is essential to start developing children’s registries too
Research training including “library” time to complete research would benefit in longer fellowships
Teaching methodology in preparation for returning to teaching/lecturing would similarly also benefit from longer fellowships
2 Training levels**: **Appendix 3 Basic competence level 1 This would involve basic concepts of PN including preventative PN and specifically AKI diagnosis and treatment using improvised techniques were not available. Ideally this could be done by previously graduated IPNA/ISN Fellows so that the “Trainee fellows become the teachers” and ensure adequate training in level 1 basic competence for 6–12 months Advanced level 2 Fellows could then proceed to level 2 Advanced Skills training for a further 12–18 months to develop more advanced skills in not only AKI but also kidney biopsies and histology, CKD, dialysis, and transplantation (optional) (Level 1 basic competence and 2 advanced clinical skills training syllabus for PN training—devised by Yap/McCulloch for IPNA.) IPNA website
Post training visits by mentors to PN fellows’ home institution for 2–4 weeks to offer support and advocacy, encourage nurses, multidisciplinary teams, and junior doctors to become enthusiastic about paediatric nephrology
Sister centers as developed by IPNA/ISN/IPTA—development of emerging and supportive centers following training of IPNA/ISN/ISPD fellows who then return to their home institution and are motivated to set up PN programs at these institutions where there may or not have been such programs The aim is for these emerging centers to team with supportive centers (often from higher resourced regions) preferably from a similar geographic region to assist in educational development including teaching, development of protocols and services not previously well developed Tension not to provide finances for drugs and service delivery as this may end up being a never-ending challenge
Adult nephrology fellows to spend some time in paediatric units to learn skills in managing children with kidney diseases especially in regions where this may not be available Visa versa it also benefits PN fellows to spend time in adult units once the COVID pandemic abates
Advocacy in particular for management of children’s kidney diseases including access for both acute and chronic dialysis not only at hospital level but also at local and national government levels
Learning about fundraising and meeting up with charities to promote access of paediatric patients to appropriate equipment and facilities for kidney disease
Continuing Medical Education (CME) by virtual teaching including Journal clubs
WhatsApp (or similar) to support groups of children’s kidney specialists to manage difficult cases

### Social factor consideration

The training of PN fellows funded by IPNA/ISN/ISPD and APFP has resulted in fellows not only becoming teachers and leaders in their own institutions/universities, but also leaders in IPNA, even as councilors.

All the PN Fellows who have trained at RCWMCH deserve significant accolades for the sacrifices of family time (many up to 24 months) they have made to gain knowledge and training to return to their home countries with skills to support paediatric nephrology. Funding to allow visits back home should be seen as being essential considering the sacrifice these fellows make. Without the commitment of these fellows, African PN would be a poorer specialty. Following the initiation of a PN training program at RCWMCH, PN units in the Gauteng region (Johannesburg and Pretoria) and more recently Durban have also expanded this training program for fellows from other parts of Africa with the same dedication of the participants that we have witnessed.

## Conclusion

This paediatric nephrology fellowship “based in Africa for Africa” has made it possible for physicians to get comprehensive PN clinical training as a result of their commitment, the commitment of those at RCWMCH and the funding support from multiple organizations. The collaborative experience has contributed to substantial improvement in the availability of pediatric kidney care in LMICs.

### Current IPNA fellows training situation

Since the IPNA Fellowship Program was initiated almost 20 years ago, more than 260 fellows have completed their training, coming from more than 56 countries, most of them low-income countries. Currently, 41 training centers participate in this initiative, aimed to disseminate pediatric nephrology expertise to under-served areas of the world. During the current year, 13 fellows are being trained in centers located in South Africa, China, Singapore, France, and Brazil, and 14 fellows are about to start their training, half of them coming from the African continent, highlighting the relevance of this program for the region (personal communication Francisco Cano March 2023).

## Supplementary Information

Below is the link to the electronic supplementary material.

Graphical abstract (PPTX 49 KB)

Appendices 1–9 (DOCX 49 KB)

## Data Availability

The datasets generated during and/or analyzed during the current study are available from the corresponding author on reasonable request.
