# Supplementary material for: Lessons learned from regional training of paediatric nephrology fellows in Africa
Source: Pediatr Nephrol. 2023 Jun 6;38(11):3757–68. doi: 10.1007/s00467-023-06022-9 (PMC10243235; doi:10.1007/s00467-023-06022-9)
Supplement: Supplementary file 1 — Graphical abstract (PPTX 49 KB) [file 467_2023_6022_MOESM1_ESM.pptx]

## Slide 1
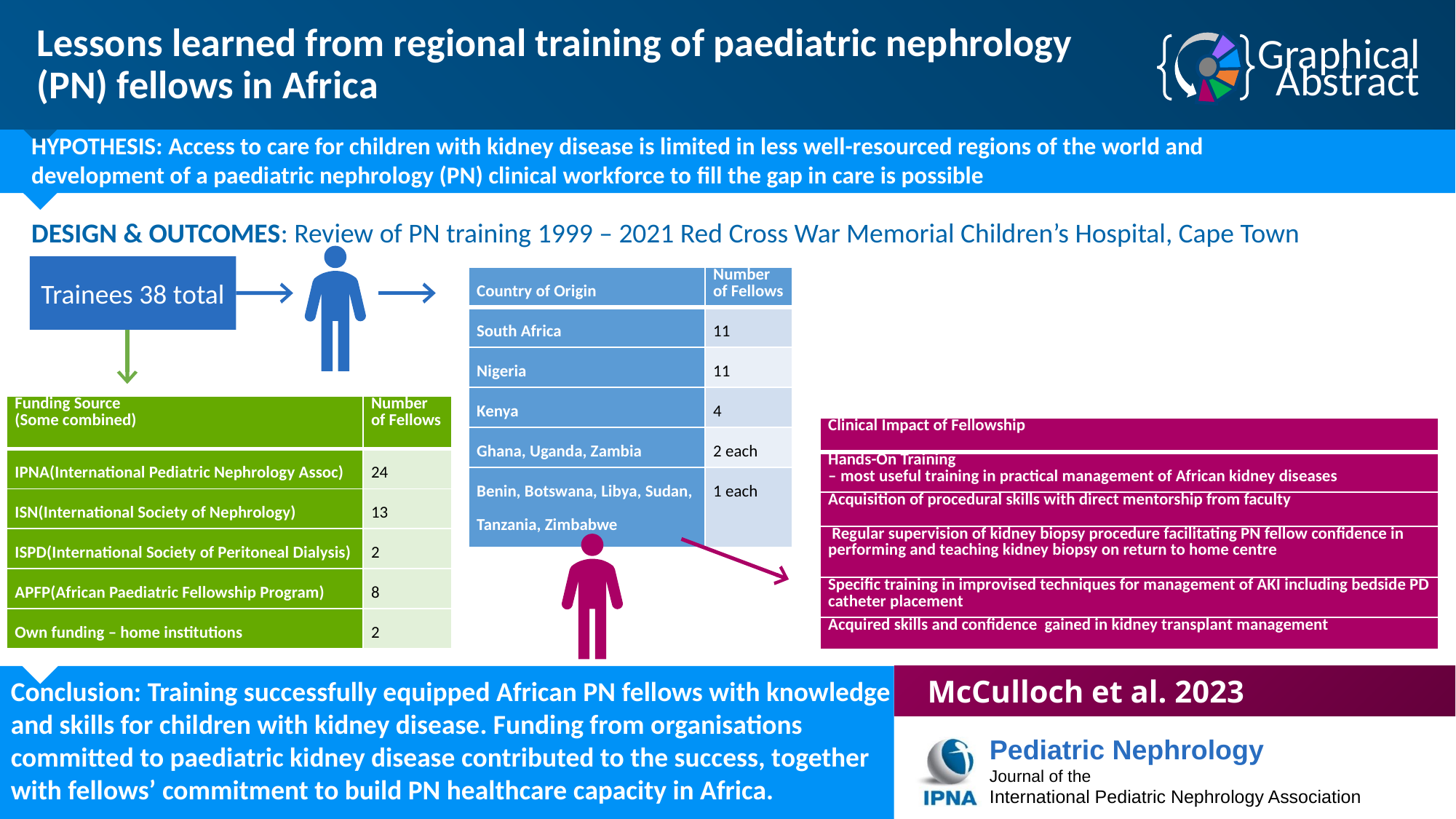

Lessons learned from regional training of paediatric nephrology
(PN) fellows in Africa
HYPOTHESIS: Access to care for children with kidney disease is limited in less well-resourced regions of the world and development of a paediatric nephrology (PN) clinical workforce to fill the gap in care is possible
DESIGN & OUTCOMES: Review of PN training 1999 – 2021 Red Cross War Memorial Children’s Hospital, Cape Town
Trainees 38 total
| Country of Origin | Number of Fellows |
| --- | --- |
| South Africa | 11 |
| Nigeria | 11 |
| Kenya | 4 |
| Ghana, Uganda, Zambia | 2 each |
| Benin, Botswana, Libya, Sudan, Tanzania, Zimbabwe | 1 each |
| Funding Source (Some combined) | Number of Fellows |
| --- | --- |
| IPNA(International Pediatric Nephrology Assoc) | 24 |
| ISN(International Society of Nephrology) | 13 |
| ISPD(International Society of Peritoneal Dialysis) | 2 |
| APFP(African Paediatric Fellowship Program) | 8 |
| Own funding – home institutions | 2 |
| Clinical Impact of Fellowship |
| --- |
| Hands-On Training – most useful training in practical management of African kidney diseases |
| Acquisition of procedural skills with direct mentorship from faculty |
| Regular supervision of kidney biopsy procedure facilitating PN fellow confidence in performing and teaching kidney biopsy on return to home centre |
| Specific training in improvised techniques for management of AKI including bedside PD catheter placement |
| Acquired skills and confidence gained in kidney transplant management |
McCulloch et al. 2023
Conclusion: Training successfully equipped African PN fellows with knowledge and skills for children with kidney disease. Funding from organisations committed to paediatric kidney disease contributed to the success, together with fellows’ commitment to build PN healthcare capacity in Africa.
