# Supplementary material for: Lessons learned from regional training of paediatric nephrology fellows in Africa
Source: Pediatr Nephrol. 2023 Jun 6;38(11):3757–68. doi: 10.1007/s00467-023-06022-9 (PMC10243235; doi:10.1007/s00467-023-06022-9)
Supplement: Supplementary file 2 — Appendices 1–9 (DOCX 49 KB) [file 467_2023_6022_MOESM2_ESM.docx]

**Supplement**

**Appendix 1 Context of Training Appendix 2 Fellows’ Survey Feedback form**

**Appendix 3 Syllabus for training at RCWMCH**

**Appendix 4 Paediatric Nephrology Level 1 and 2 training**

**Appendix 5 Fellows trained for Africa**

**Appendix 6 Daily Workload on return home return including clinical, teaching, and administrative commitments**

**Appendix 7 Work location at home institution**

**Appendix 8 Recommended Modifications to the Training Program**

**Appendix 9 Fellows Responses to Training Experience**

**Appendix 1 Context of Training**

| **Hospital environment** | | |
| --- | --- | --- |
| Red Cross War Memorial Children’s Hospital | Children’s hospital which provides multiple levels of care from primary care (to community in close proximity); general paediatric care; specialist and subspecialist services including most paediatric and paediatric surgical subdisciplines. Close access to paediatric imaging services (radiology, ultrasonography, CT and MRI scans, angiography, cardiac catheterization, nuclear medicine) – both diagnostic and interventional. Direct access to laboratory services (haematological, microbiological, histopathological, immunological, pharmacological, research and clinical) | This hospital is equipped with many of the resources that would be available in high income countries, but not in most LMICs.  The hospital services are deeply integrated within the healthcare systems of the Western Cape Provincial Department of Health and the City of Cape Town  Transport services are in place to transfer emergency patients from around the province, and from other regions of the country |
| Groote Schuur Hospital Adolescent | Large tertiary hospital which provides in-patient and out-patient services for adolescents. The clinical services at the hospital include all specialist and subspecialist services for adults. As the hospital includes large obstetric and neonatal service, there are large groups of neonates (providing access to kidney issues in neonates). There are also specialist services that focus on the transitioning of care for adolescents and young adults | This hospital has many of the resources available in high income countries. The experience in this institution helps to expose trainees to the overlaps between adult and paediatric services across the world |
| Tygerberg Children’s Paediatric Nephrology | This tertiary hospital does not provide transplantation services, nor cardiac surgical services for children. However, the patient’s spectrum in this hospital is closer to what trainees are likely to experience in their own countries. | Patient base is more clinic based with referrals from LMIC environments. The hospital does have access to high income country resources and does refer patients requiring specialist dialysis services as well as transplantation to the Red Cross War Memorial Children’s Hospital. Follow up services also include children who have been treated at the RCWMCH in the past. |
| **Specific areas of exposure** | | |
| RCWMCH, TCH and GSH | General pathology | African specific pathology |
| General nephrology | Urinary tract infections, Enuresis, Glomerulonephritis, HUS, Hypertension | Acute Post Streptococcal Glomerulonephritis |
| Urodynamics services | Advanced laboratory services at the RCWMCH enable detailed studies and analysis of bladder functions |  |
| Laboratory services | The clinical services are supported by laboratory services from the National Health Laboratory services. | Uniquely the services have access to specialists in paediatric pathologies. This ranges from chemical laboratory services (which have a specialist interest in inborn errors of metabolism; in the development of markers for severe disease in children); to paediatric pathology and histopathology services (which are able to provide a wide range of services including light microscopy, electron microscopy and a wide range of specialist “labelling services” which enable the identification of particular immunological processes. |
| Paediatric urology services | The paediatric nephrology and urology services are closely integrated, and they share access to the urodynamics services. | Obstructive and Reflux pathology, VACTERL; Late presenting posterior urethral valves |
| Chronic Dialysis program for Chronic Kidney Disease (CKD) | There is a dedicated team that provides both Peritoneal dialysis and Haemodialysis for patients in chronic kidney failure | These services are primarily directed at supporting children who would be candidates for kidney transplantation. Generally, patients would not be accepted onto chronic kidney replacement therapy unless they were potential transplant candidates. This service provides a clinical exposure, but also provides a setting for the development of ethical processes for prioritization of expensive modalities of therapy. |
| Acute Renal support services | The acute renal support services are available 24 hours, 7 days per week to provide a wide range of acute renal support services including: peritoneal dialysis, haemofiltration (different modalities), haemodialysis, plasmapheresis | These services are provided through a unique private public partnership, which means that trainees are exposed to the services, but they are also exposed to the options for underlying systems that enable this sort of service. |
| Transplantation | The RCWMCH and the GSH both provide transplantation services (liver, kidney, heart, stem cell, lung – at GSH). The surgical teams (while primarily based at the RCWMCH) do work across the platform, and they share services such as the transplant co-ordination for identification and management of potential donors. Tissue typing and immunological testing is available through the service. | The trainees are exposed to transplantation services; the processes of identifying a procuring potential donors; the services required etc.  An additional component of this experience is the presence of a palliative care programme, which provides support for patients and families where dialysis / transplantation is not being offered.  The exposure to both solid organ and haematological transplant services expose trainees to multidimensional aspects of transplantation. |
| Neonates and Antenatal care | Congenital anomalies of kidney and urinary tract (CAKUT) | While antenatal screening is limited, there are patients with prenatally diagnosed kidney abnormalities that present to the services. The institutions have access to clinical and laboratory-based genetics services that allow fully workup of patients with congenital anomalies. |
| PICU (Paeds Intensive care Unit) | There are 2 paediatric intensive care units within the system. The PICU at the RCWMCH provides a multidisciplinary critical care service to a wide range of disciplines including paediatrics; paediatric subspecialities; cardiothoracic services; paediatric and neonatal surgery; burns; trauma, etc | The PICU provides an environment where complex critical care can be provided to patients from a wide variety of disciplines. Trainees are exposed to management of kidney disease in emergency and elective surgical patients. The PICU is able to support multiple modes of acute and chronic kidney replacement therapy. |
| Rheumatology services (from paediatric through adolescent and adult services | The overlap between some rheumatological conditions and paediatric nephrology may be significant, and these services are closely interlinked | Particular experience with Takayasu’s arteritis and its many manifestations has been achieved.  Other vasculitides including systemic lupus erythematosus are managed jointly by team. |
| Paediatric Sub-specialty services | Oncology, Cardiology, Pulmonology, Endocrine | Often presenting late or end stage, Hodgkin’s, Cystinosis |
| Infectious Diseases | The infectious disease and infection control teams work closely with the nephrology teams. There is additional overlap between clinical immunology and the infectious disease teams. There is research laboratory services related to these disciplines which play an important role in training. | Conditions such as HIV and Tuberculosis, are commonly seen in the Western Cape, and may have multiple implications for paediatric nephrology services.  While tropical conditions such as Malaria and Schistosomiasis are infrequently seen in the Western Cape, there is some exposure to these conditions as well.  The infectious disease team also plays an important role in the development and implementation of antibiotic management programmes in the institutions. |
|  |  |  |
| **University** | | |
| Universities of Stellenbosch and Cape Town | All the training takes place in the contexts of the Health Sciences faculties of the Universities. This means that there is a solid basis and support structure for the development of research training and support, as well as training in disciplines related to paediatric nephrology. |  |
| **STAFF COMPONENT** | | |
| Senior Clinicians – paediatric nephrologists - are available at all times according to a rota in terms of supervision and hands -on training for any nephrology, dialysis, PICU and transplant support and teaching.  Paediatric trained nurses and dialysis technologists, urodynamic technologists and  allied health including paediatric dietitians, social workers, psychologists, and physiotherapists. | | |
| Relevant Staff to nephrology include Paediatric urology and surgery; Paediatric anesthesia, pathologist and paediatric radiologists | | |
| Facilities – Laboratory services – Haematology, Biochemistry, Microbiology, Histology; Radiology including MRI, Screening, Nuclear Medicine and MRI, Blood bank | | |
| Inpatient Facilities - Paediatric Intensive Care Unit, Renal Unit with High Care, Emergency Unit | | |
| Outpatient clinics – both Renal and Urology clinics | | |

**Appendix 2**

**Fellows Survey Feedback Form**

Name:

Fellowship dates:

Fellowship site/s:

Mentor:

Current position held including academic & leadership position:

Position prior to fellowship:

Current workplace/s:

Contact Address

Mobile/WhatsApp no:

Email address:

Any other relevant information

Thank you for training with us and we are proud of the work you are doing in your home institution. Please could you complete this voluntary questionnaire

1. How much time are you spending doing paeds nephrology?

<10%_____/10-25%______/25-50%_____/50-70%_______/100%______

1. Where are you working?

State__________/University____________/Private_______________

1. How much of your time is:
   1. Clinical__________________________________________________________
   2. Teaching_______________ (if so who are you teaching)__________________
   3. Administrative____________________________________________________
2. What areas/skills of training specifically were useful in the context that you work in?

(e.g. clinical skills; management skills; research skills; administrative processes; computer and database related issues.

1. Was it useful to your setting to have exposure to the following and why?
   1. Specialist nurses and clinical technologists and why?
   2. Other specialties and which in particular?
   3. Different modalities of dialysis and relevant to your setting?
2. What areas/skills need improvement?
3. Was Acute Kidney Injury (AKI) management covered?
4. Have you been able to do Peritoneal Dialysis in your current practice?

If so:

Number per year?

What catheters have you used?

Manufactured fluid/Home-made?

If not performed what has been the barriers?

1. Have you been able to do Haemodialysis?

HD in adult unit?

If not performed what has been the barriers?

1. Have you been able to perform other forms of CRRT and which machines are available?

If not performed what has been the barriers?

1. Was the hands-on training useful and in what way?
2. Did you spend time at another institution – details including length of time and benefits?
3. Did you complete a post graduate nephrology examination (Diploma/UCT or Certificate via College of Medicine)?

If you have, how has it been helpful?

If not what were the barriers

1. Does your institution require a paediatric nephrology qualification when you return from training?
2. Have you been able to do any research in the field of paediatric nephrology and if so brief outline?

Where you able to complete an MPhil?

If not, what were the barriers

1. Have you attended any Nephrology Congresses or courses? Paediatric or Adult?

Were you able to present any papers/posters/invited lectures?

1. Does your country have a Paeds or Renal society and do they support paeds nephro?
2. Has the home institution been supportive to you? In what way?

Have they assisted in acquisition of equipment?

What equipment has been most useful to you?

1. Do you have access to the following supportive services and what are the challenges with each? Are they available during office hours and also at night/weekends?
   1. Radiology
   2. Histopathology
   3. Surgery/Urology
2. What other specific work-related challenges on return home are an issue?

e.g., Job structure, supporting personnel, salaries, access to hospital beds/ICU beds. Salaries and posts, IT services and hospital records, senior and junior staff support, admin support etc.

1. Has health care in your community been affected by your training?

e.g. Have more nephrological problems been identified, Have people taken on new approaches to management of renal issues, Is it the clinical training, the people skills, the administrative and management issues that have been changed

1. Are you involved in any teaching formally or informally?

Did your training prepare you for this?

1. Do you participate in CME activities?

What?

1. What additional CME would be useful?
2. What areas/skills of training need to be modified for future trainees?
3. Would you recommend the program to interested individuals and why?
4. Any other comments?

Thanks for taking the time to complete this.

Best wishes

Mignon McCulloch

**Appendix 3**

**Syllabus for training at RCWMCH**

TRAINING PROGRAM IN PAEDIATRIC NEPHROLOGY RED CROSS WAR MEMORIAL CHILDREN’S HOSPITAL(RCWMCH)

INDEX:

- CURRICULUM
  - Aim
  - Core topics covered
  - Skills to be acquired
    - Patient management
    - Practical experience
    - Research & presentations
- RENAL UNIT TIMETABLE
  - Details of patient profile which teaching Unit manages
  - Details of available auxiliary facilities/services which are available to the Teaching Unit
  - Physical facilities and equipment available

FELLOWS ROLE IN NEPHROLOGY SERVICE

- - **Service duties:** Working in nephrology ward helping to manage patients post-transplant on acute and chronic dialysis as well as general nephrology.
  - **OPD:** helping to manage outpatients in general nephrology and transplant
  - **After hours:** One weekend call a month as well one week call per week. This is always done with consultant as second on call. The aim of this is mainly for teaching because much of the nephrology work occurs after hours.
  - **Research:** The Fellow is expected to participate in research activities.
  - **Training:** Fellows are expected to assist in training of undergraduate as well as registrars in paediatric nephrology

CURRICULUM FOR PAEDIATRIC NEPHROLOGY TRAINING AT RED CROSS WAR MEMORIAL CHILDREN’S HOSPITAL(RCWMCH)

AIM

Providing a qualified Paediatrician with adequate hands-on and theoretical Paediatric Nephrology skills to practice Paediatric Nephrology in an educated, safe and appropriate manner.

##### CORE TOPICS COVERED

- Urinary tract infections(UTI’s)
- Nephrotic syndrome (e.g. Minimal Change, Mesangioproliferative, Mesangiocapillary and Focal Segmental glomerulosclerosis).
- Glomerulonephritis including post-infective nephritis
- Acute Kidney Injury(AKI) – related to shock and sepsis including gastroenteritis, post-operative cardiac surgery and drug related causes
- Chronic Kidney Disease(CKD)
- Paediatric Hypertension – diagnosis and management
- Haemolytic Uraemic Syndrome(HUS) – especially diarrhoea associated
- Renal Ricketts and Metabolic Bone Disease
- Paediatric Vasculitides e.g. Polyarteritis Nodosa, Takayasu’s Disease.
- Autoimmune conditions such as Systemic Lupus Erythematosus requiring immunosuppression
- Antenatal abnormalities – follow-up of congenital renal abnormalities problems detected antenatally
- Paediatric Urologic problems – posterior urethral valves, pelvi-ureteric junction obstructions
- Kidney related endocrine problems - Diabetes Insipidus
- Kidney support in Paediatric Intensive Care Patients (PICU)
- Kidney support in other specialties e.g., Paediatric oncology
- Adolescent Paediatric Kidney Disease
- Paediatric dialysis including peritoneal, haemodialysis, CVVH and Plasmapheresis
- Transplantation – pre, intra-operative and post-operative management including management of immunosuppression
- Paediatric kidney palliative care

**SKILLS TO BE ACQUIRED:**

###### PATIENT MANAGEMENT

Ward based

- Clinical assessment of patients with kidney diseases including BP, weight, fluid status and urine dipsticks
- Day to day management of general Kidney, Dialysis and Transplant patients including admission and assessment, ordering of investigations, performing procedures and management decisions.
- Management of kidney referrals from other wards
- Advising on management of kidney problems in patients in the Paediatric ICU including initiation of dialysis (both Peritoneal and CRRT)
- Accompanying patients to theatre who are having Tenckhoffs placed as well as those going for renal transplants
- Liaising closely with other specialist services at the hospital
- Telephonic consultations and email responses to paediatricians and GP’s outside of the hospital
- Working closely with a multi-disciplinary team
- Acquiring hands-on skills related to procedures such as dialysis(catheters and prescriptions) and kidney biopsies
- Presenting interesting cases at academic meetings
- Management of Adolescents with renal disease.

Outpatients

- Management and follow up of children with all kidney pathologies attending renal clinic and presenting as day cases.
- Outreach peripheral clinics and teaching
- Telephonic referrals from further afield in South Africa

Acquisition of skills required to maintain a renal unit including:

- Reports & Statistics - keeping accurate patient records, formulating reports and completing audits related to patient care.
- Equipment – Maintaining adequate supplies of disposable and accessing permanent equipment.
- Ethical kidney issues and patient rights
- Patient and parent counseling
- Kidney teaching to Students and Registrars

#### PRACTICAL EXPERIENCE

Acute Peritoneal Dialysis Insertion of Acute and Semi-permanent PD catheters at bedside – from neonates through to adolescents

(Weight range 900gm to 100kg)

Ordering dialysis prescription for both manual &

automated cycling peritoneal dialysis

Managing complications of peritoneal dialysis

Chronic PD Accompanying patient to theatre to ensure correct

placement of Tenckhoff PD catheters and assistance to surgical insertion team

Initiate prescription of chronic dialysis

Long term monitoring of adequacy of dialysis using peritoneal equilibration testing (PET) as follow up.

Acute Haemodialysis Setting up Haemodialysis and CRRT machine in the Renal unit as well as in the PICU

Ordering dialysis prescription for individual patients

Supervising training of nursing staff

Managing complications of haemodialysis/CVVH/CVVHD using Fresenius Multifiltrate machines

Plasmapheresis including indications and prescription

Neonatal Dialysis using Carpe Diem

Chronic HD Chronic Kidney Support mainly including all indications and complications associated

Assessment of Chronic HD lines and fistulas

Renal Biopsy Acquiring skills to perform safe and adequate renal biopsies under sedation with Ultrasound (real time) guidance

.

Histology Weekly combined renal biopsy reviews

PICU Kidney care Managing Medical/Surgical/Post transplant patients with kidney problems – advising and performing dialysis

Transplantation Assist with pre-, peri and post operation care and work up of all paediatric transplant patients. Management of patients on Immunosuppressive therapy including various immunosuppressive regimens and monitoring.

Urodynamics Understanding the techniques involved in paediatric urodynamics/video-urodynamics as well as being able to interpret results and make clinical decisions based on the results

#### RESEARCH & PRESENTATIONS

Clinical Audit and Research Facilitate research, publication and abstract development is encouraged.

Presentations Case presentations on ward rounds, at multidisciplinary meetings in the hospital and at combined adult meetings. Local Research day presentation. Journal club and combined meetings.

#### RENAL UNIT TIMETABLE

| Week at Red Cross |
| --- |
| Monday  8-9am Peritoneal dialysis clinic  9-1pm Ward work / Ward Attenders  11am Dialysis ward round  12pm Social round  2-5pm Renal Ward Round |
| Tuesday  8-9am Surgical Meeting  9-10am Urology Round  10.10-11am X-ray meeting  11-12pm Ward Round – Fellow facilitated  1-4pm Renal Clinic |
| Wednesday  8-9am Academic meeting  9-11 am Kidney biopsies  9 – 1pm Ward work  12pm Teaching ward round  2-3pm Journal club |
| Thursday  8-11am Ward work  11-12pm Subspecialty Ward Round  2 – 5pm Afternoon Research/Admin alternating with other Fellows  2 – 4pm Adolescent clinic at GSH (adult hospital) (3rd/month) |
| Friday  8 – 12pm Renal Clinic  12-1pm D3 medical meeting case presentations  1 – 4pm Ward round/ward work |
| Week divided into Ward duties, Dayward and dialysis, Office and consult |
| After hours: One week day call per week ; one weekend per month on call with consultant as part of the learning program |

## Details Of Patient Profile Which Teaching Unit Manages

Age:

Premature infants and newborns through to adolescents

Demographics:

Local patients as well as referrals from all over country as well as other African countries

Private and State patient mix

The unit manages children and adolescents with renal diseases including the following:

**1) Glomerular Diseases**

a. Primary

i. FSGS

ii. Membranous nephropathy

iii. Mesangiocapillary

iv. IgA Nephropathy

v. Vasculitides causing glomerulopathies e.g., SLE, Wegener’s, HSP

b. Secondary

i. Post infective Nephritis

ii. HIV related renal diseases

iii. Others…

2) **Congenital kidney abnormalities** such as

a. Prune belly

b. PUJ VUJ obstruction

c. Posterior urethral valves

d. Vesico-ureteric reflux

e. Bladder extrophy

f. Dysfunctional voiding

g. Neurogenic bladder

3) **Developmental kidney abnormalities**

a. Renal dysplasia –cystic/ non cystic

4) **Cystic Kidney Diseases**

a. ADPKD

b. ARPKD

c. Other cystic diseases

5) **Hereditary Kidney Diseases**

a. Nephronophthisis

b. Alport syndrome

c. Primary hyperoxalurias

d. Cystinosis

e. Renal tubular acidosis

f. Nephrogenic Diabetes insipidus

g. Barrter and Gitelmans syndromes

h. Mitochondrial cytopathies

**6) Arterial Hypertension**

a. Investigation and management of

**7)** **Acute Kidney Injury**

**8)** **Chronic Kidney Disease**

Secondary to above cause

**9) Dialysis**

Acute

Chronic

Peritoneal /Haemodialysis /CVVH/Plasmapheresis

**10) Renal Transplantation**

One of South Africa’s 2 active Paediatric Renal Transplant units:

o Including small children as little as 10kg

o Also living related renal transplantation from adults to children

o Total 271 kidney transplants since 1968

**Appendix 4**

**Level 1 – Basic Competence**

- Fundamental knowledge – kidney structure & function, blood & electrolyte results with relevant management
- Clinical examination & assessment skills
- Assessment & management of ped nephrology problems in inpatient and out-patient settings
- Knowledge of fluid & electrolyte imbalance in renal pathology
  - Urinalysis
  - Fluid resuscitation (Oral and IV, Different fluids available)
- Understanding basic renal imaging techniques for UTI’s
- Understanding social implications of CKD
- Principles of prescribing medication in children with renal dysfunction
- Knowledge of basic disorders
  - Hematuria & Proteinuria
  - UTI’s
  - Nephrotic & Nephritic
  - AKI and CKD
  - Hypertension
  - Renal Calculi
- Outpatient Department follow-up of patients with renal pathology
- Develop communication skills with respect to the care of children with kidney diseases
- Manage the patient with acute kidney injury
  - Conservatively
  - Peritoneal dialysis, if possible
- Perform small audit project
- Knowledge of screening and awareness of paediatric renal disease
- Proficiency in preventive nephrology

**Level 2 Clinical Skills**

- Outpatient attendance under supervision
  - Management of general nephrology/urology patients (Transplant – optional)
  - Growth and nutrition in children with renal disease
- Ward admission and management of complicated paediatric nephrology patients
  - Acute new patients including AKI
  - Ward responsibility with evaluation of urinalysis, checking and interpreting blood results, phoning parents, keeping the files current with latest results, etc.
  - First-line on-call roster after hours
  - Managing CKD and chronic dialysis patients
- Interpretation of radiologic, ultrasound, nuclear and urodynamic studies
- Presenting cases on ward rounds, and grand/academic teaching rounds
- Perform practical procedures under supervision including
  - Renal biopsies
  - Dialysis related, such as insertion of acute peritoneal dialysis catheters
  - Observing or assisting in insertion of hemocatheters
- Acquire knowledge of renal histology by attending histology meetings

**Appendix 5 Fellows Trained for Africa**

**IPNA/ISN/ISPD/APFP FUNDED PAEDS NEPH FELLOWS FROM AFRICA WORKING AT RCWMCH 2003 - 2021**

| **Gender** | **Origin** | **Date Training completed** | **Time spent** | **Funding** | **Master or Diploma** |
| --- | --- | --- | --- | --- | --- |
| F | Nigeria | March 2004 | 6months | IPNA |  |
| M | Uganda | Sept 2004 | 6mths | IPNA/ISN |  |
| M | Kenya | Aug 2007 | 12mths | IPNA/ISN |  |
| M | Kenya | Aug 2007 | 15mths | IPNA/ISN |  |
| M | Benin | Jan 2010 | 18mths | IPNA/ISN | Cert Ped Neph |
| M | Nigeria | Feb 2008 | 18mths | IPNA/ISPD  APFP | Cert Paed Neph, MPhil/MPH |
| M | Ghana | May 2009 | 19mths | Ghana hospital  IPNA | Cert Paed Neph, MPhil |
| M | Kenya | July 2010 | 12mths | IPNA/ISN |  |
| M | Nigeria | June 2010 | 6mths | IPNA |  |
| M | Kenya | Aug 2011 | 12mths | IPNA/APFP |  |
| M | Nigeria | July 2012 | 15mths | IPNA/ISN |  |
| F | Nigeria | Nov 2011 | 6mths | IPNA |  |
| M | Sudan | April 2013 | 18mths | IPNA/ APFP |  |
| F | Nigeria | Dec 2012 | 6mths | IPNA |  |
| F | Nigeria | Dec 2014 | 16mths | IPNA/ISN | PHD in process |
| F | Nigeria | March 2014 | 6mths | IPNA |  |
| F | Nigeria | Oct 2014 | 22mths | IPNA/ISN/  APFP | Cert Paed Neph  MPhil |
| F | Zambia | Dec 2015 | 24mths | Beit Trust | Cert Paed Neph  MPhil |
| M | Tanzania | Dec 2015 | 12mths | IPNA/ISN | PG Diploma in Ped Nep |
| F | South Africa | March 2018 | 12mths | IPNA | PG Diploma in Paeds Neph |
| F | Nigeria | Sept 2018 | 11mths | IPNA | PG Diploma in Paeds Neph |
| F | Uganda | Dec 2019 | 28mths | ISN/APFP | Cert Paed Neph  MPhil |
| F | Ghana | Sept 2019 | 25mths | IPNA/  APFP | Cert Paed Neph  MPhil |
| M | Nigeria | Dec 2018 | 3mths | ISPD |  |
| F | Zimbabwe | Dec 2021 | 23mths | IPNA/ISN APFP | Cert Paed Neph  MPhil |
| F | Botswana | Dec 2021 | 23mths | IPNA/ISN/ APFP | Cert Paed Neph  MPhil |
| F | Libya | Dec 2021 | 27mths | IPNA | Cert Paed Neph  MPhil |
| M | Zambia | June 2021 | 24mths | ISN | Cert Paed Neph |

**Appendix 6 Daily Workload on Return to Home Institution**

|  | |
| --- | --- |
| Time allocation | Number of fellows (%) |
| **Clinical Time** | |
| *>50%* | *15(63%)* |
| 25 – 50% | 8(33%) |
| <25% | 1(4%) |
| **Teaching Time** | |
| *25 – 50%* | *10(48%)* |
| 15 – 25% | 7(33%) |
| >50% | 4(19%) |
| **Administration Time** |  |
| *10 – 25%* | *11(50%)* |
| 25 – 50% | 5(23%) |
| <10% | 5(23%) |
| >50% | 1(4%) |

**Appendix 7 Work Location at Home Institution**

| **Work Facility** | |
| --- | --- |
| Work Base | Number of fellows (%) |
| State hospitals (Government) | 9 (33%) |
| University hospitals | 8 (29%) |
| State and Private hospitals combination | 5 (18%) |
| State and University hospitals combination | 2 (8%) |
| University and Private hospitals combination | 1 (4%) |
| State/University and Private hospitals combination | 1 (4%) |
| Private hospitals only | 1 (4%) |

**Appendix 8 Recommended Modifications to the Training Program**

| *Return of fellows every 18-24months for a 4 week period to refresh skills* |
| --- |
| *Visiting of Trainers to centres to assist in training* |
| *More haemodialysis to be learnt and fixing of HD catheters* |
| *Training to teach and educational methodologies* |
| *More Research time* |
| *Vascular access skills* |
| *Urodynamics development and teaching* |
| *Return as a Transplant fellow for a year to spend time in HLA lab or Histopathology* |
| *Time in intensive care unit for extra training* |

**Appendix 9 Fellows Responses to Training Experience**

| *Most definitely – practical hands-on fellowship training with similar scenarios to home town* |
| --- |
| *Definitely. This was the best training I ever had. The immersion was perfect. Everyone was supportive. The cases were those we meet at home. We had the opportunity to really practice, not just only to observe.* |
| *Yes. The exposure at RXH is very good since there is a great amount of input from all disciplines especially during the grand rounds. The training at RXH was also hands on. I remember the very first day I was asked to do ward round in RXH and I was to green when it comes to nephrology. But it helped me to grow.* |
| *I strongly recommend the programme. The similarities with the practice in one’s home country and the hands-on nature make it worth all the value* |
| *Yes. The program provides a solid foundation in paediatric nephrology upon which trainees can build specifically much needed capacity for the specialty in resource constrained environments.* |
| *RXH offers quaternary care. Need support staff and histopathologists to go for training. High volumes of patients and need to train more PN* |
| *Definitely, it was a real transformative period in my medical career and practice. The hands-on nature of the training is very commendable and empowering.* |
| *YES!!!! This training is very broad since it does not just cover clinical skills but also administration, research advocacy. It’s a very good program.* |
| *Yes: Paediatric Nephrology is still in its infancy/non- existent in most parts of Africa and therefore, to improve the outcomes of renal conditions, we need to train more people* |
| *A very comprehensive teaching and academic program with excellent experienced mentors and seniors. Supportive environment: mentors are very supportive and caring. All of us as fellow felt like being with a family away from home.* |
